# Micro-RNA-155 is induced by K-Ras oncogenic signal and promotes ROS stress in pancreatic cancer

**DOI:** 10.18632/oncotarget.4125

**Published:** 2015-05-12

**Authors:** Peng Wang, Chao-feng Zhu, Ming-zhe Ma, Gang Chen, Ming Song, Zhao-lei Zeng, Wen-hua Lu, Jing Yang, Shijun Wen, Paul J. Chiao, Yumin Hu, Peng Huang

**Affiliations:** ^1^ Sun Yat-sen University Cancer Center, State Key Laboratory of Oncology in South China, Collaborative Innovation Center of Cancer Medicine, Guangzhou, China; ^2^ Department of Emergency Medicine, Sun Yat-sen Memorial Hospital, Guangzhou, China; ^3^ Department of Translational Molecular Pathology, The University of Texas MD Anderson Cancer Center, Houston, TX, USA; ^4^ Department of Molecular and Cellular Oncology, The University of Texas MD Anderson Cancer Center, Houston, TX, USA

**Keywords:** K-Ras, miR-155, reactive oxygen species, pancreatic cancer

## Abstract

The oncogenic K-Ras can transform various mammalian cells and plays a critical role in development of pancreatic cancer. MicroRNAs (miRNA) have been shown to contribute to tumorigenic progression. However, the nature of miRNAs involved in K-Ras transformation remains to be investigated. Here, by using microarray we identified miR-155 as the most upregulated miRNA after both acute and prolonged activation of K-Ras in a doxycyline-inducible system. Pharmacological inhibition of MAPK and NF-κB pathway blocked the induction of miR-155 in response to K-Ras activation. Overexpression of miR-155 caused inhibition of Foxo3a, leading to decrease of major antioxidants including SOD2 and catalase, and enhanced pancreatic cell proliferation induced by ROS generation. Importantly, correlations of K-Ras, miR-155 and Foxo3a were also validated in human pancreatic cancer tissues. Therefore, we propose that miR-155 plays an important role in oncogenic K-Ras transformation mediated by cellular redox regulation.

## INTRODUCTION

Oncogenic mutations of the K-Ras gene are present in >90% of pancreatic ductal carcinoma [1], which is one of the most aggressive and deadly cancer [2]. In most cases, the K-Ras mutations found in cancer cells introduce amino acid substitution at positions 12 and 13 [3]. The K-Ras^G12V^ mutations are frequently detected in precursor lesion to pancreatic cancer, pancreatic intraepithelial neoplasia (PanIN), indicating an important role of K-Ras in early development of pancreatic cancer [2, 4–6].

Activating K-Ras mutations are highly associated with disease progression, due to activation of several effector proteins and downstream pathways that induce cell proliferation, survival and invasion [7, 8], including Raf kinase, phosphatidylinositol 3′-kinase, and RalGDS proteins. [3, 9, 10]. While these effector pathways exert major roles in Ras transformation, other downstream factors such as reactive oxygen species have been suggested to contribute to Ras transformation potential [11]. Moderate increase of ROS can stimulate cell growth and proliferation due to their role as messengers in cellular signal transduction pathway, thus, contribute to tumor growth and cancer development [12]. Growing evidence suggests that cancer cells, compared with normal cells, produce high levels of ROS and hence are constantly under oxidative stress [13, 14]. Indeed, our previous study using doxycline-inducible system indicated that the K-Ras^G12V^ mutations caused significant elevation of ROS production through a yet unknown mechanism [15].

MicroRNAs (miRNA) are a class of small noncoding RNAs comprising approximately 22 nucleotides in length [16] and play critical roles in many biological processes by directly interacting with specific messenger RNAs (mRNAs) through base pairing and then inhibiting expression of the target genes [16]. MicroRNAs can act as oncogenes or tumor suppressor genes and miRNA expression is deregulated in cancer by a variety of mechanisms including amplification, deletion, mutation, and epigenetic silencing [17]. This led us to investigate whether miRNA is responsible for *K-Ras* oncogenic transformation and to further study the association between miRNA and redox regulation by K-Ras activation.

In the current study, we report the identification of miR-155 as the most upregulated miRNA after K-Ras activation in doxycycline-inducible system and suggest that miR-155 is a candidate oncogenic miRNA in pancreatic cancer. Our results showed that K-Ras upregulated miR-155 expression through MAPK and NF-κB pathway and miR-155 promoted pancreatic cancer cell proliferation by increase of ROS levels through inhibiting Foxo3a expression. Our findings reveal miR-155 as a mechanistic link between K-Ras oncogenic signal and redox regulation.

## RESULTS

### MiR-155 is specifically upregulated after K-Ras activation in doxycycline-inducible K-RasG12V expression cell system

**Figure 1:**
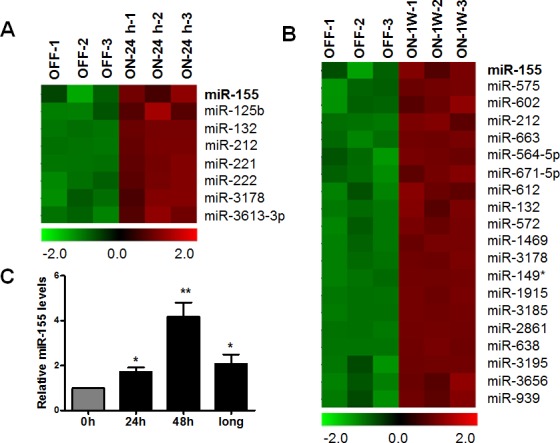
Increase of miR-155 expression after K-Ras activation in T-Rex/K-Ras cells **A.**–**B.** MiRNA array data analysis of upregulated miRNAs in T-Rex/K-Ras cells after K-Ras activation induced by 100 ng/ml doxycyline. OFF, control cells without K-Ras activation. ON-24h, cells with K-ras activation for 24 hrs. ON-1W, cells with K-Ras activation for 1 week. *n* = 3, *P* < 0.05. **C.** MiR-155 expression was detected by qRT-PCR after K-Ras activation by 100 ng/ml doxycycline for 24 hrs (24h), 48 hrs (48h) and more than 1 month (long). Data are shown as mean ± SD, *P < 0.05; **P < 0.01, *n* = 3.

We have previously established a doxycycline-inducible K-Ras^G12V^ expression cell system (designated as T-Rex/K-Ras cells) to directly test the potential effect of oncogenic activation of K-Ras to the downstream signaling pathways [15]. To investigate whether miRNAs are responsible for *K-Ras* oncogenic transformation, miRNA array analysis was used to detect the differentially expressed miRNAs after activation of K-ras. The doxycycline-inducible system enables control of defined experimental conditions to allow a precise time-course study of correlation between K-Ras activation and the changes of the downstream molecular events. Indeed, the addition of doxycyline induces a time-dependent expression of K-Ras protein as described before [15]. As the microarray results shown in Figure 1A, 8 miRNAs were highly expressed after short term induction of K-ras for 24 hrs. 20 miRNAs were highly expressed after long term induction of K-ras for 1 week. Interestingly, miR-155 was most upregulated after both short term and long term induction of K-ras.

To further validate the microarray results, qRT-PCR assay was used to evaluate miR-155 levels in T-Rex/K-ras cells after K-ras activation at different time points. As shown in Figure 1C, miR-155 showed approximately 2 fold and 4 fold increase after K-Ras activation for 24 and 48 hrs. Moreover, miR-155 remained significantly upregulated after long term induction of K-ras for more than 1 month, suggesting that elevation of miR-155 was not merely a stress response to acute K-Ras activation.

### K-Ras stimulates miR-155 expression through MAPK and NF-κB pathway

**Figure 2:**
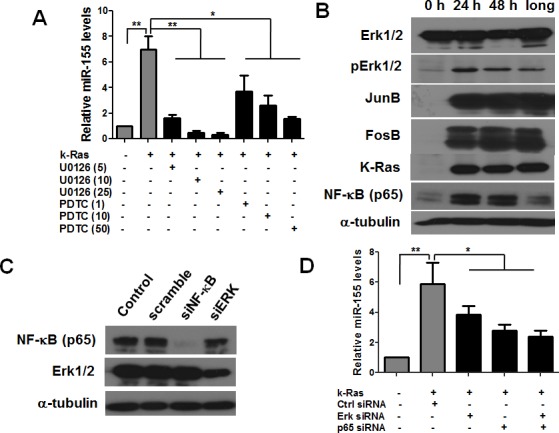
K-Ras induces miR-155 expression through MAPK and NF-κB pathway in T-Rex/K-Ras cells **A.** qRT-PCR data showing increased miR-155 levels after K-ras activation for 48 hrs and such increase was reversed by ERK1/2 inhibitor U0126 and NF-κB (p65) inhibitor PDTC at various concentrations (μM). Data are shown as mean ± SD,*P < 0.05; **P < 0.01, *n* = 3. **B.** Protein expression of pERK1/2, JunB and FosB in MAPK pathway and NF-κB (p65) expression after activation (24 and 48hrs) and long term activation (>1 month) of K-Ras. α-tubulin was used as loading control. **C.** Protein expressions of NF-κB (p65) and ERK1/2 were significantly inhibited by siRNA. α-tubulin was used as loading control. **D.** qRT-PCR data showing increased miR-155 levels after K-ras activation for 48 hrs and such increase was reversed by inhibiting the protein expression of NF-κB and ERK by siRNA. Data are shown as mean ± SD, *p < 0.05; **p < 0.01, *n* = 3.

We then investigated the downstream signaling pathway that links K-Ras to miR-155 expression. We found that the augmented expression of miR-155 after K-Ras activation was significantly inhibited by an ERK inhibitor U0126 in a dose-dependent manner and a similar inhibitory effect by an NF-κB inhibitor PDTC, which indicated the critical roles of activated MAPK and NF-κB in K-Ras induced miR-155 expression (Figure 2A). Indeed, MAPK activity was found to be markedly elevated as demonstrated by significant increase of phosphorylated Erk after K-ras was activated by doxycyline for 24 hrs, 48 hs and more than one month (Figure 2B). Consistently, the downstream transcription factor JunB and FosB were also upregulated. Similarly, NF-κB p65 was also significantly upregulated after K-Ras activation (Figure 2B). To further test whether MAPK and NF-κB pathway mediate K-Ras induced expression of miR-155, T-Rex/K-Ras cells with K-Ras activation for 24 hrs were transfected with Erk and NF-κB (p65) specific siRNAs. The knockdown of Erk and NF-κB was verified by western blot analysis as shown in Figure 2C. Transfection of siErk and siNF-κB (p65) significantly suppressed the increase of miR-155 expression induced by K-ras activation. In contrast, control siRNA showed no inhibitory effect of miR-155 expression (Figure 2D). AP1 is a heterodimeric transcription factor which can be formed by JunB and FosB. Our results were in consistence with previous studies that miR-155 promoter contains an AP-1 active site and an NF-κB site [18, 19].

In addition to the doxycycline-inducible T-Rex/K-Ras cell model, we also tested miR-155 expression in K-Ras transformed human pancreatic ductal epithelial cells established previously [20]. The human pancreatic ductal epithelial cells HPDE and hTERT-HPNE cells transfected with K-Ras exhibited approximately 3 and 6 fold increase of miR-155 expression compared with the parental cells (Figure S1A). Two naturally occurring pancreatic cancer cell lines with K-ras mutation including Capan-2 (G12V) and Aspc-1 (G12D) were also used to test the effect of K-Ras and the downs stream effectors on miR-155 expression. As shown in Figure S1B–C, specific knockdown of K-Ras, Erk and NF-κB in both cell lines significantly suppressed the expression of miR-155. Taken together, our results suggest that oncogenic K-Ras induced the miR-155 expression through the NF-κB and MAPK-AP1 pathway.

### MiR-155 mediates ROS generation by inhibition of Foxo3a

**Figure 3:**
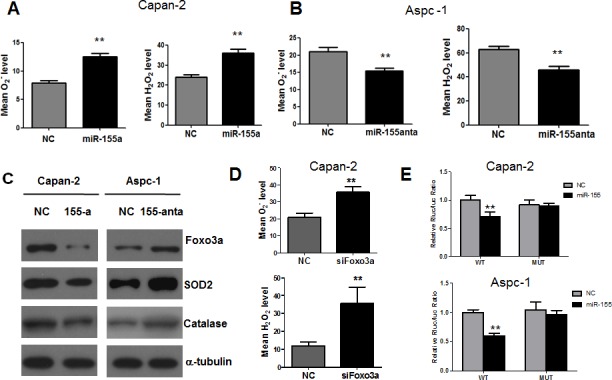
MiR-155 causes inhibition of Foxo3a and subsequent increase of ROS generation in pancreatic cancer cells **A.** Measurement of superoxide (O_2_^−^, detected by fluorescent probe HEt) and hydrogen peroxide (H_2_O_2_, detected by fluorescent probe DCF-DA) levels in Capan-2 cells before and after transfection of miR-155 micmics. **B.** Measurement of O_2_^−^ and H_2_O_2_ levels in Aspc-1 cells before and after transfection of miR-155 antagomir. **C.** Capan-2 cells were transfected with miR-155 mimics and Aspc-1 cells were transfected miR-155 antagomir. The indicated protein expression was compared before and after transfection. α-tubulin was used as loading control. **D.** Measurement of O_2_^−^ and H_2_O_2_ levels in Capan-2 cells before and after inhibition of Foxo3a by siRNA. **E.** Luciferase activity in pancreatic cancer cells co-transfected with miR-155 and pmiR-RB-REPORT dual luciferase reporter plasmid. WT, wild type FOXO3a-3′-UTR region. MUT, mutated FOXO3a-3′-UTR region. All data are shown as means ± SD, **P < 0.01, *n* = 3.

Increased ROS stress has been observed in a wide spectrum of human cancers and has been shown to be associated with oncogenic signals such as c-myc and Ras [21, 22]. Indeed, our previous study using the doxycycline inducible T-Rex/K-Ras cell model also demonstrated that activation of K-Ras induced rapid increase of ROS levels in a time-dependent manner [15]. It is known that ROS may serve as messengers and increase of ROS may promote cancer cell proliferation and contribute to cancer development. This prompted us to investigate whether miR-155 mediates K-ras transformation through redox regulation. We found that miR-155 expression was relatively lower in Capan-2 compared with Aspc-1 (Figure S2A). MiR-155 agomir (miR-155a) was transfected into Capan-2 cells and antagomir (miR-155anta) was transfected into Aspc-1 cells and the effect of transfection was tested in these cell lines. Overexpression of miR-155 in Capan-2 cells caused significant increase in both superoxide (O_2_^−^) and hydrogen (H_2_O_2_) levels (Figure 3A). In contrast, inhibition of miR-155 in Aspc-1 cells caused significant decrease in both O_2_^−^ and H_2_O_2_ levels (Figure 3B).

We then investigated the mechanism by which miR-155 caused ROS accumulation. We searched for the potential target genes of miR-155 in TargetScan database and identified FOXO3 as a candidate target (Figure S2B). Indeed, the interaction between miR-155 and FOXO3a has been suggested in breast cancer cells [23]. In consistence, we found a higher miR-155 level and a lower Foxo3a level in Aspc-1 cells compared with Capan-2 cells, indicating an inverse link between miR-155 and Foxo3a (Figure S2A). As shown in Figure 3C, transfection of miR-155 mimics suppressed FOXO3a expression in Capan-2 cells and miR-155 antagomir caused increase of FOXO3a expression in Aspc-1 cells, which confirmed the inverse relationship between miR-155 and FOXO3a expressions. Knockdown of Foxo3a indeed induced significant accumulation of both O_2_^−^ and H_2_O_2_ in Capan-2 cells (Figure 3D). Antioxidant enzymes play essential roles in protecting cells against ROS insults. We postulated that miR-155 may induce ROS generation through regulation of antioxidant expression. Indeed, the inhibition of FOXO3a by miR-155 mimics caused down regulation of SOD2, a major antioxidant that converts mitochondrial O_2_^−^ to H_2_O_2_. Catalase, another major antioxidant that eliminates H_2_O_2_ was also decreased after transfection of miR-155 mimics in Capan-2 cells (Figure 3C). In contrast, transfection of miR-155 antagomir caused increase of FOXO3a expression, leading to upregulation of both SOD2 and catalase (Figure 3C). A luciferase reporter assay was then performed to test the direct binding of miR-155 to FOXO3a (Figure 3E). The potential miR-155 binding sequence in the 3′-UTR region of FOXO3a was cloned into a luciferase reporter vector and co-transfected with miR-155 into Capan-2 and Aspc-1 cells. Vector with mutated 3′-UTR region of FOXO3a was also generated a negative control. As shown in Figure 3E, overexpression of miR-155 caused 30-50% decrease of luciferase activity in both Capan-2 and Aspc-1 cells transfected with wild type 3′-UTR region of FOXO3a. In contrast, miR-155 did not affect the luciferase activity in both cell lines transfected with mutated 3′-UTR region of FOXO3a, indicating direct binding of miR-155 to FOXO3a in pancreatic cancer cells.

**Figure 4:**
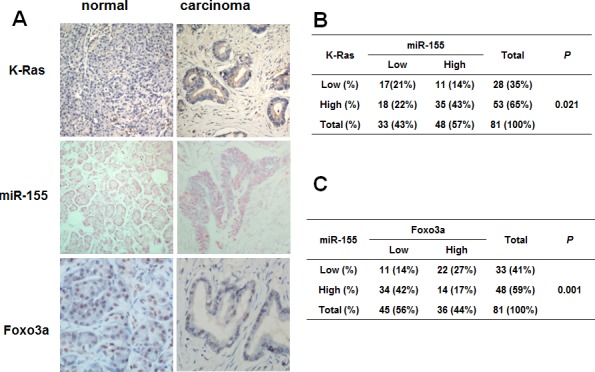
Correlation of K-Ras, miR-155 and Foxo3a expression in human pancreatic cancer tissues **A.** Representative tissue microarray showing immunostaining of K-Ras and Foxo3a and in situ hybridization of miR-155 in human pancreatic ductal carcinoma and normal pancreatic ducts (original magnification, 200×). **B.** Association between K-Ras and miR-155 expression in 81 pancreatic cancer tissue samples, *P* = 0.021 **C.** Association between miR-155 and Foxo3a expression in 81 pancreatic cancer tissue samples, *P* = 0.001. The above tissue microarray staining results were analyzed by Fisher's exact test.

To test the clinical relevance of the above observations, we analyzed the expression of K-Ras, miR-155 and FOXO3a and their correlation in primary pancreatic tissue microarray containing 81 cases of pancreatic ductal carcinoma samples and the matched normal pancreatic tissues. As illustrated in Figure 4A, cancer tissues exhibited stronger immunostaining signals of both K-ras and miR-155 compared with the normal tissues. In contrast, cancer tissues exhibited decreased FOXO3a levels compared with normal tissues. In addition, we observed a significantly positive correlation between K-ras and miR-155 expression and a significantly inverse correlation between miR-155 and FOXO3a expression in pancreatic cancer tissues. Taken together, our results suggested that miR-155 may play an important role in redox regulation as a downstream effector of K-Ras.

### MiR-155 promotes pancreatic cancer cell proliferation through ROS accumulation

**Figure 5:**
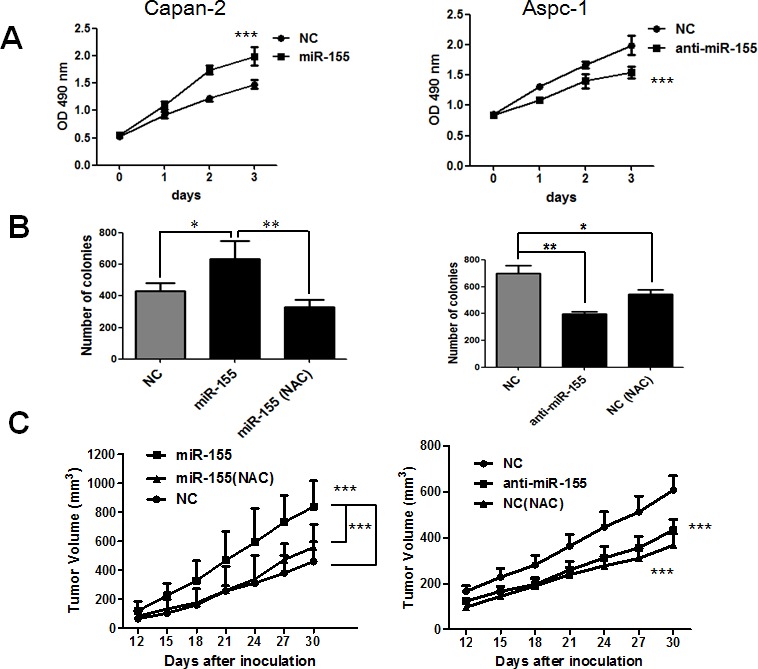
MiR-155 promotes pancreatic cancer proliferation through ROS generation *in vitro* and *in vivo* **A.** Comparison of cell proliferation before and after stable transfection of miR-155 mimics in Capan-2 cells (left panel) and miR-155 antagomir in Aspc-1 cells (right panel). Cell proliferation was analyzed by MTS assay. *P* < 0.001, *n* = 3. **B.** Quantitative comparison of colony formation before and after stable transfection of miR-155 mimics in Capan-2 cells (left panel) and miR-155 antagomir in Aspc-1 cells (right panel). Capan-2 with miR-155 mimics and Aspc-1 control cells were also incubated with 1mM NAC to test its effect on colony formation. **C.** Left panel, the first group of 6 mice were inoculated subcutaneously with 2×10^6^ Capan-2 cells, whereas the second group of 6 mice were inoculated with same number of Capan-2 cells stably transfected with miR-155 mimics. The third group of 6 mice were inoculated with same number of Capan-2 cells stably transfected with miR-155 mimics and received water supplement with 60mM NAC. Right panel, the first group of 6 mice were inoculated subcutaneously with 2×10^6^ Aspc-1 cells, whereas the second group of 6 mice were inoculated with same number of Aspc-1 cells stably transfected with miR-155 antagomir. The third group of 6 mice were inoculated with same number of Aspc-1 cells and received water supplement with 60mM NAC. Tumor size was measured at the indicated time points after inoculation and expressed as mean volume±SD. *P* < 0.001, *n* = 6.

To test the role of miR-155 induced ROS production in pancreatic cancer cell survival, we stably transfected miR-155 mimics in Capan-2 cells and miR-155 antagomir in Aspc-1 cells using lentiviral particles. The overexpression of miR-155 led to significant increase in cell proliferation and colony formation capacity in Capan-2 cells (Figure 5A and 5B). Importantly, the enhanced colony formation capacity by miR-155 was reversed by treatment with ROS scavenger NAC (Figure 5B). To test the effect of miR155 on tumor growth *in vivo*, nude mice were divided into three groups (*n* = 6 in each groups). The first group of mice was subcutaneously inoculated with 2×10^6^ Capan-2 cells and the second one was inoculated with same number of Capan-2 cells with miR-155 mimics. The third group was inoculated with Capan-2/miR-155 mimic cells and given standard water supplemented with 60 mM NAC (N-acetyl cysteine). As shown in Figure 5C, transfection of miR-155 significantly promoted tumor growth of Capan-2 cells (*P* < 0.001). Importantly, in consistence with the *in vitro* result, such acceleration of tumor growth by miR-155 was also rescued by supplement with 60 mM NAC (*P* < 0.001). In contrast to the effect of overexpression, inhibition of miR-155 in Aspc-1 cells suppressed the pancreatic cancer cell proliferation both *in vitro* and *in vivo* (Figure 5A–5C, right panel).

## DISCUSSION

Aberrant expression of miroRNAs has been observed in a wide spectrum of malignancies and contributes to tumorigenesis and disease progression. However, the underlying mechanism of deregulation of miRNAs and its association with oncogenic transformation remains to be investigated. K-Ras represents the most frequently mutated gene found in solid tumors and occurs in more than 90% pancreatic cancer. We have previously established a doxycycline inducible cell system with K-Ras^G12V^ expression vector (T-Rex/K-Ras cells) [15]. The defined experimental conditions doxycycline inducible system enables a precise study of temporal correlation between K-Ras activation and alteration of downstream molecular events.

The miRNA array results identified 8 and 20 highly expressed miRNAs respectively after short term (24 hrs) and long term (1 week) activation of K-Ras. Interestingly, miR-155 was the most upregulated miRNA in both rapid and prolonged induction of K-Ras. Dysregulation of miR-155 levels has been associated with various types of cancers, including lung cancer, breast cancer, colon cancer and lymphatic system [24, 25], indicating the prominent role of miR-155 in cancer biology. The influence of oncogenic signals to the most commonly overexpressed miRNA in malignancies remained to be determined. Here, we have shown that oncogenic K-Ras constitutively up-regulates expression of miR-155 through the MAPK and NF-κB pathway.

We then further investigated the consequence of miR-155 alteration in the context of K-Ras oncogenic signal. Using the doxycycline inducible cell system with K-ras^G12V^, we previously discovered that K-ras activation leads to tow major metabolic alterations including metabolic switch from oxidative phosphorylation to glycolysis and substantial accumulation of oxidative stress [15]. The current study showed that ectopic expression of miR-155 targets Foxo3a, leading to decrease of two major antioxidants including SOD2 and catalase. SOD2 is a mitochondrial enzyme that converts O_2_^−^ to H_2_O_2_ and catalase is responsible for eliminating H_2_O_2_ and protecting cells from ROS insults. Cellular ROS generation is counterbalanced by the action of antioxidant enzymes to eliminate the harmful effect of ROS. Overexpression of miR-155 indeed caused elevation of both O_2_^−^ and H_2_O_2_ as a result of inhibition of Foxo3a. It is worth noting that we previously found that K-Ras activation in the doxycycline inducible system caused substantial decrease of both SOD2 and catalase expression, although the underlying mechanism remained to be determined [15]. Mir-155 may be a mechanistic link responsible for the intrinsic oxidative stress induced by K-ras oncogenic signal.

It is interesting to note that, in certain experimental model, K-Ras appeared to lower ROS level through increased transcription of Nrf2 (NF-E2-related factor 2) [26], a critical transcription factor of a number of major antioxidants that protect cells from oxidative stress. In this study, DeNicola *et al*. used an endogenous and conditional oncogenic LSL-KRasG12D alle and demonstrated lower ROS level of K-RasG12D/+ MEFs compared with K-RasLSL/+ MEFs due to increase of Nrf2, which leads to more reduced intracellular environment. Interestingly, we did not observe significant increase of Nrf2 expression in response to K-Ras induction or miR-155 overexpression in the current study (data not shown). In spite of the appearing contradicting results from different experimental conditions, elevated levels of endogenous oxidative stress has been observed in many cancer cell types and *in vivo* [27–29]. Cancer cells that survive ROS stress are likely to acquire adaptive mechanisms and enhance antioxidant capability to counteract the potential toxic effect of ROS. Indeed, activation of redox adaptation including redox-sensitive transcription factor and upregulation of antioxidant molecules has been observed in various cancers [29–32]. As such, activation of Nrf2 in certain experimental model may also reflect such adaptation mechanism *in vivo* in response to ROS stress. The current study of doxycyline inducible system with ectopic K-RasG12V expression may represent the initial increase of ROS induced by acute induction of K-Ras. In addition, regulation of redox balance by different mutation of K-Ras (G12D and G12V) may also require further investigation.

**Figure 6:**
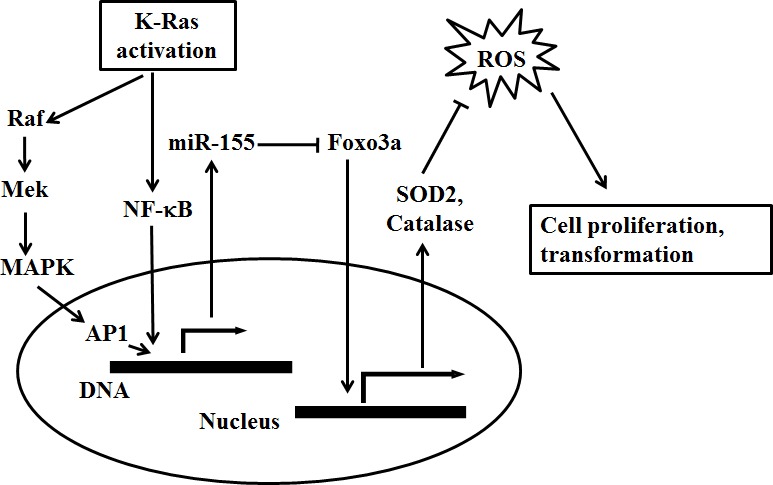
A proposed model for the role of miR-155 in mediating K-Ras induced ROS generation and cell proliferation in pancreatic cancer Oncogenic activation of K-Ras stimulates miR-155 expression via MAPK and NF-κB pathway. MiR-155 negatively regulates the expression of its downstream target Foxo3a, a transcription factor that promotes the expression of two major antioxidants including SOD2 and catalase. As such, enhanced expression of miR-155 by oncogenic K-Ras may result in further inhibition of Foxo3a, leading to decrease of SOD2 and catalase and subsequent accumulation of ROS stress that contribute to cell transformation and cancer cell proliferation.

In summary, the present findings identify up-regulation of miR-155 as a consequence of exposure to K-Ras oncogenic signal, which is constitutively activated in more than 90% pancreatic cancer. As illustrated in Figure 6, our study gives rise to a possibility that miR-155 may play a mediating role in oncogene induced ROS stress and neoplastic growth of pancreatic cancer.

## MATERIALS AND METHODS

### Reagents

The following antibodies were used for immunoblotting analyses using standard western blotting procedures: α-tubulin, ERK, p-ERK, JunB, FosB and NF-κB (Cell Signaling Technology); Foxo3a and SOD2 (Epitomics); K-Ras (Abcam); Catalase (Santa Cruz). ERK1/2 inhibitor was purchased from Promega. NF-κB inhibitor and ROS scavenger NAC were purchased from Sigma.

### Cell culture

The doxycycline inducible T-Rex/K-Ras cells were constructed as previous described [15] and cultured in Dulbecco's modified Eagle's medium supplemented with 10% tetracycline free FBS. The pancreatic cancer cell lines (Capan2, Aspc1, Panc1 and BxPC3) were purchased from American Type Culture Collection. The human pancreatic duct epithelial cell lines (HPDE and HPNE) and K-Ras transformed human pancreatic duct epithelial cell lines (HPDE/K-Ras and HPNE/K-Ras) were cultured in DMEM/F12 medium supplemented with 10% FBS as described before [33].

### MiRNA microarray analysis

Total RNA of T-Rex/K-Ras cells before and after K-ras activation was extracted using Trizol Reagent (Invitrogen) according to manufacturer's instructions. Microarray assay and data analysis were provided by LC Sciences, Houston, TX. The experiments were performed in triplicates. Data were analyzed by subtracting the background and normalizing the signals using a LOWESS filter.

### Relative quantitative reverse transcription-polymerase chain reaction (qRT-PCR)

Total RNA was isolated using Trizol (Invitrogen) according to the manufacturer's instructions. MiRNA was reverse-transcribed using All-in-One™ miRNA qRT-PCR Detection Kit (GeneCopoeia). Real-time PCR analysis was performed using the SYBR Premix Ex Taq II kit (TaKaRa) and detected by Bio-Rad detection system (Bio-Rad). Human U6 snRNA was used as endogenous control for quantitation of miRNAs.

### ROS detection

The cells were stained with 5 μM CM-H_2_DCFDA (Invitrogen, for H_2_O_2_ detection) or 100 ng/ml dihydroethidium (Sigma, for O_2_^−^ detection) for 60 minutes to detect cellular ROS. Cells were then collected and washed with PBS and measured by flow cytometer (FC500, Beckman Coulter).

### Cell transfection and lentivirus infection

The small interfering RNAs (siRNAs) target for *ERK1/2*, *NF-*κ*B*, *Foxo3a* were synthesized by Cell Signaling Technology. MiR-155 agomir or antagomir, and control RNA duplex (NC) were synthesized by RiboBio (Guangzou, China). Cells (∼70% confluent) were transfected with 100 nM miRNA agomir, antagomir, siRNA or NC using Lipofectamine™ RNAiMAX (Invitrogen) according to manufacturer's instructions. The lentivirus (GenePharma) was used to construct stable cell lines transfected with miR-155 mimics or inhibitors using the Lenti-Pac™ HIV Expression Packaging Kit (GeneCopoeia). The cells without miR-155 mimics or inhibitors transfection was used as a negative control (NC).

### MTS assay and colony formation assay

Cells were seeded into 96-well plates and cultured for 72 hrs. 20 μl of MTS solution (Promega, Madison, WI) was added into each well and cells were incubated at 37°C for 3 hs before absorbance was detected at 490 nm with a microplate reader (SpectraMax M5, Molecular Devices). Same number of cells were seeded in six-well plates and cultured for 14 days. Colonies were fixed with methanol for 10 minutes and stained with crystal violet solution for 30 minutes.

### Immunohistochemistry

The expression of K-Ras and Foxo3a was detected by immunohistochemical staining on pancreatic cancer tissue microarray (Shanghai Outdo Biotech, China) with 81 pancreatic ductal carcinoma and their paired non-neoplastic pancreatic tissue samples. The slide was incubated with primary antibodies against K-Ras (Abcam, 1:200 dilution) or Foxo3a (Epitomics, 1:200 dilution) overnight at 4°C. The score of immunohistochemical staining was performed as described previously [34].

### *In situ* hybridization of miRNA

The expression of miR-155 was detected by in situ hybridization on human pancreatic cancer tissue microarray (Shanghai Outdo Biotech, China). For the detection of the miR-155, DIG labeled LNA-miR-155 probe (Exiqon) was used according to manufacturer's instructions. MiR-155 expression was visualized by anti-DIG-AP conjugate antibody and NBT/BCIP substrate (Roche). The staining score was performed as described previously [34].

### Luciferase assay

DNA fragments from 3′-UTR of FOXO3a containing the predicted complementary sites of miR-155 were cloned to downstream of the Renilla luciferase reporter gene in pmiR-RB-REPORT dual luciferase reporter plasmid (RiboBio, Guangzhou, China). The predicted 3′-UTR of FOXO3a was also mutated as a negative control to test the direct binding of miR-155 to FOXO3a. The sequence of FOXO3a 3′-UTR were designed as follows: 5′-AGCATTA-3′ (wild type, WT) and 5′-TCGTAAT-3′ (mutation, MUT). Capan2 and Aspc1 cells overexpressed with miR-155 were transfected with plasmid containing wild type or mutated fragments from 3′-UTR of FOXO3a by Lipofectamine LTX (Life Technologies). Dual luciferase signals were measured 48 hours after transfection by the Dual-luciferase assay kit (Promega).

### Tumor xenograft model

Animal studies were approved by the Institutional Animal Care and Use Committee of Sun Yat-sen University. 2×10^6^ cells were subcutaneously injected onto one flank of 5-week old female nude mice. The mice were divided into 6 groups with 6 mice in each group: (1) Capan-2 xengograft. (2) Capan-2/miR-155 mimic xenograft. (3) Capan-1/miR-155 mimic xenograft supplemented with 60 mM NAC in water. (4) Aspc-1 xenograft. (5) Aspc-1/miR-155 agomir xenograft. (6) Aspc-1 xenograft supplemented with 60 mM NAC in water. Tumor volume was measured every 3 days and calculated using the equation: volume (mm^3^) = ( length×width^2^)/2.

### Statistical analysis

The correlation between K-Ras, miR-155 and Foxo3a expression in tissue microarray was performed by Fisher's exact tests. The statistical difference in pancreatic cancer cell growth in vitro and in vivo between different groups was analyzed by repeated measures AOVA. All other analyses were performed by two-tailed Student's t test. The data shown are means ± SD. *P* < 0.05 was considered statistically significant.

## SUPPLEMENTARY MATERIAL FIGURES


