# Supplementary material for: Micro-RNA-155 is induced by K-Ras oncogenic signal and promotes ROS stress in pancreatic cancer
Source: Oncotarget. 2015 May 12;6(25):21148–58. doi: 10.18632/oncotarget.4125 (PMC4673256; doi:10.18632/oncotarget.4125)
Supplement: Supplementary file 1 [file oncotarget-06-21148-s001.pdf]

Micro-RNA-155 is induced by K-Ras oncogenic signal and promotes ROS stress in pancreatic cancer

Supplementary Material

Figure S1

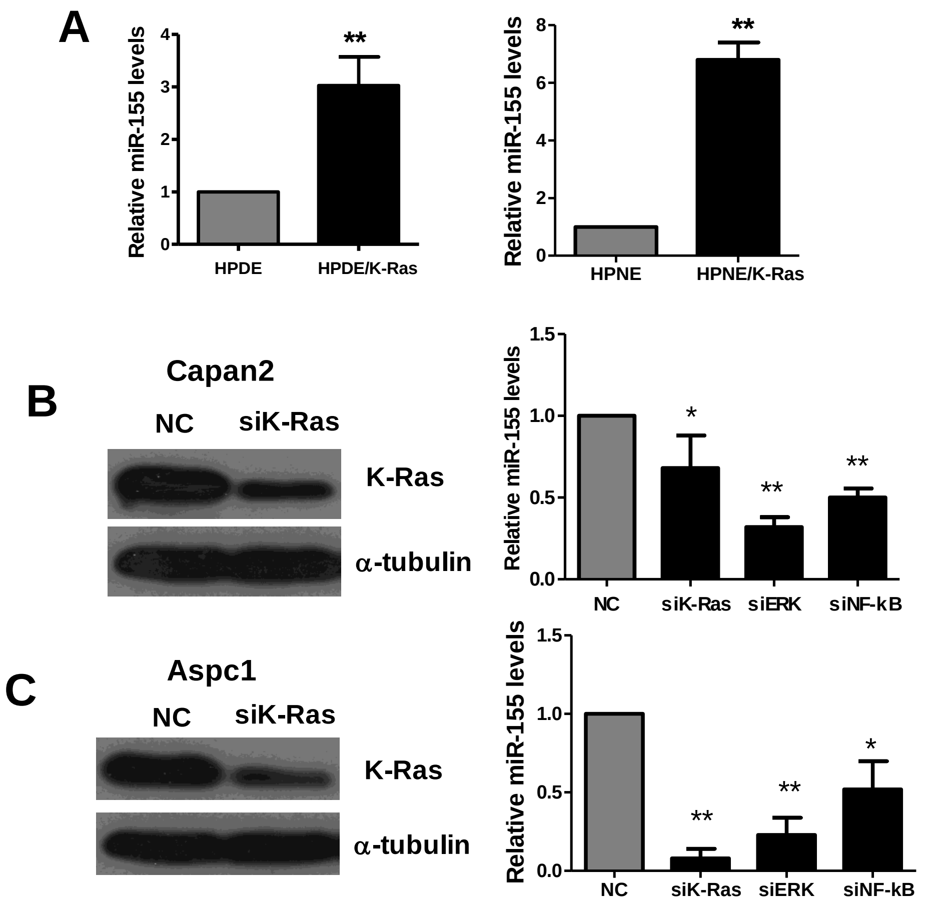

Figure S1. (A) qRT-PCR data showing substantial increase of miR-155 expression in K-ras transformed pancreatic ductal epithelial cells (HPDE/K-ras, HPNE/K-Ras) compared with the parental cells (HPDE, HPNE). Data are shown as mean ± SD, $^{**}p < 0.01$, n = 3. (B-C) Inhibition of K-Ras, ERK and NF-κB significantly inhibited miR-155 expressions in Capan-2 and Aspc-1 cells.

Figure S2

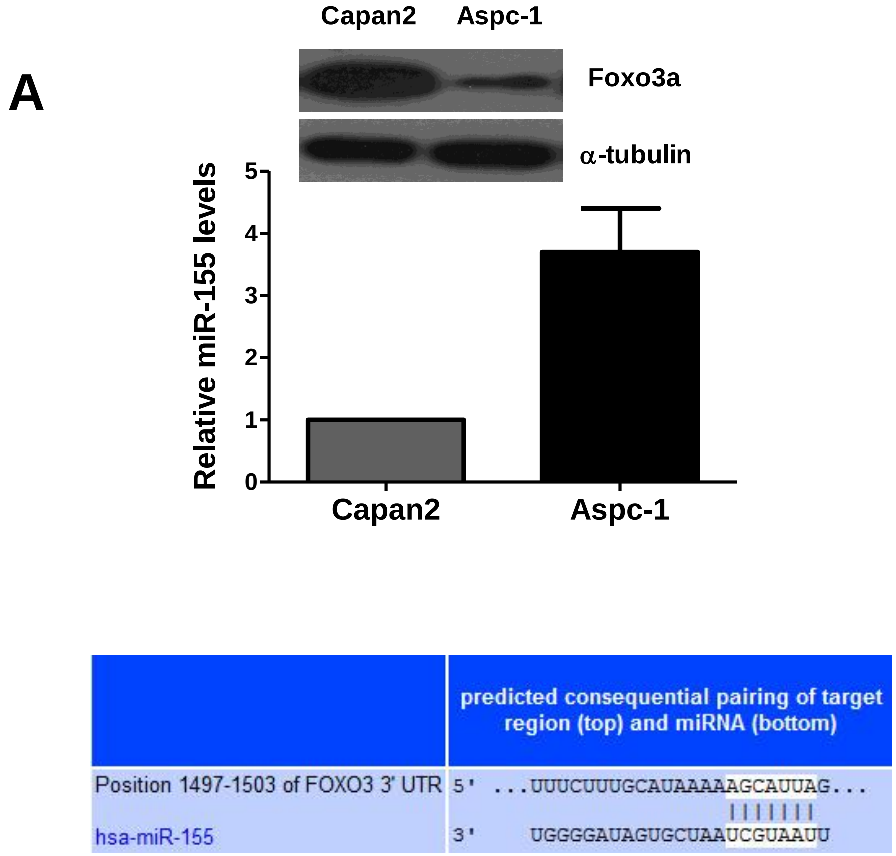

Figure S2. (A) Inverse correlation between miR-155 and Foxo3 expression in pancreatic cancer cells Capan-2 and Aspc-1. (B) TargetScan database was used to predict the binding region of miR-155 in Foxo3 3’-UTR site.
